# Open label, randomized, crossover pilot trial of high-resolution, relational, resonance-based, electroencephalic mirroring to relieve insomnia

**DOI:** 10.1002/brb3.101

**Published:** 2012-10-28

**Authors:** Charles H Tegeler, Sandhya R Kumar, Dave Conklin, Sung W Lee, Lee Gerdes, Dana P Turner, Catherine L Tegeler, Brian C Fidali, Tim T Houle

**Affiliations:** 1 Department of Neurology, Wake Forest School of MedicineWinston-Salem, North Carolina; 2 Brain State Technologies, LLCScottsdale, Arizona; 3 Department of Anesthesiology, Wake Forest School of MedicineWinston-Salem, North Carolina

**Keywords:** Biofeedback, EEG, HIRREM, insomnia, neural oscillations

## Abstract

Effective noninvasive interventions for insomnia are needed. High-resolution, relational, resonance-based, electroencephalic mirroring (HIRREM™) is a noninvasive, electroencephalography (EEG)-based method to facilitate greater client-unique, autocalibrated improvements of balance and harmony in cortical neural oscillations. This study explores using HIRREM for insomnia. Twenty subjects, with an Insomnia Severity Index (ISI) score of ≥15 (14 women, mean age 45.4, mean ISI 18.6), were enrolled in this randomized, unblinded, wait-list control, crossover, superiority study. Subjects were randomized to receive 8–12 HIRREM sessions over 3 weeks, plus usual care (HUC), or usual care alone (UC). Pre- and post-HIRREM data collection included ISI (primary outcome), and many secondary, exploratory measures (CES-D, SF-36, HR, BP, neurocognitive testing, and VAS scales). The UC group later crossed over to receive HIRREM. ISI was also repeated 4–6 weeks post-HIRREM. All subjects completed the primary intervention period. Analysis for differential change of ISI in the initial intervention period for HUC versus UC showed a drop of 10.3 points (95% CI: −13.7 to −6.9, *P* < 0.0001, standardized effect size of 2.68). Key secondary outcomes included statistically identical differential change for the crossed-over UC group, and persistence of the effect on the ISI up to > 4 weeks post-HIRREM. Differential change in the HUC group was also statistically significant for CES-D (−8.8, 95% CI: −17.5 to −0.1, *P* = 0.047), but other exploratory outcomes were not statistically significant. For all receiving HIRREM (*n* = 19), decreased high-frequency total power was seen in the bilateral temporal lobes. No adverse events were seen. This pilot clinical trial, the first using HIRREM as an intervention, suggests that HIRREM is feasible and effective for individuals having moderate-to-severe insomnia, with clinically relevant, statistically significant benefits based on differential change in the ISI. Effects persisted for 4 weeks after completion of HIRREM. Larger controlled clinical trials are warranted.

## Introduction

Insomnia is the most prevalent sleep disorder and is associated with significant psychosocial and somatic pathology. Up to 50% of the U.S. adult population reports symptoms of insomnia on a weekly basis and approximately 12% meets criteria for insomnia disorder (Ohayon 2002). Cross-sectional studies demonstrate that 40–60% of individuals with insomnia exhibit depressive symptoms (Foley et al. 1995; Ohayon et al. 1998), 10–25% may have clinical depression, and 20–30% have anxiety disorder (Ohayon and Roth 2003; Taylor et al. 2005). Chronic insomnia is associated with reduced quality of life, higher absenteeism, impaired job performance, and higher healthcare utilization (Kuppermann et al. 1995; Simon and VonKorff 1997). In a large population-based study, a linear relationship was demonstrated between insomnia prevalence and number of self-reported comorbid medical disorders (Budhiraja et al. 2011). Insomnia severity has been correlated with suicidal thinking in a clinical trial population (McCall et al. 2010).

Although these cross-sectional associations are often interpreted to suggest that a variety of pathologies can result in secondary insomnia, prospective studies have found insomnia to be a risk factor for acute myocardial infarction (Laugsand et al. 2011) and depression (Jaussent et al. 2011). In long-term follow-up of 1741 individuals who had undergone polysomnography, insomnia was found to confer an independent and significantly increased risk for mortality (Vgontzas et al. 2010). The question of how or why insomnia should be a risk factor for other pathologies likely overlaps with the question of what processes are responsible for the pathogenesis of insomnia itself. To answer one or both of these questions, conceptualizations and data from several lines of inquiry may be helpful.

The “hyperarousal” theory (Perlis et al. 1997) highlights interplay between psychological and physiological factors in the etiology and perpetuation of chronic insomnia, including increased autonomic activity (Monroe 1967; Adam et al. 1986); activation of neuroendocrine and neuroimmunological axes (Vgontzas et al. 2001; Burgos et al. 2006), and altered brain metabolism, especially during the night (Nofzinger et al. 2004). For instance, compared with normal controls, insomnia patients show significantly increased ratio of low- to high-frequency spectral power (LF/HF, sympathetic activation) of heart rate variability (Bonnet and Arand 1998), increased production of cortisol (activity of the hypothalamic–pituitary–adrenal axis) and interleukin-6 (IL-6, activation of neuroimmunological axes) (Riemann et al. 2009), and increased power in higher frequencies as measured by spectral analysis of the sleep electroencephalogram (EEG) at sleep onset (Perlis et al. 2001a) and during nonrapid eye movement (REM) sleep (Perlis et al. 2001b). Greater amplitudes, as measured by event-related EEG potentials, were observed in several latency ranges prior to, during, and on awakening (Devoto et al. 2005; Steiger 2007; Yang and Lo 2007; Bastien et al. 2008). Taken together, these data suggest that heightened cortical arousal may be either part of the pathogenesis of chronic primary insomnia or a consequence of it, or both.

Disruption of biological rhythms is another way to model the etiology and sequelae of insomnia (Reid and Zee 2009). Virtually all physiological systems function on a rhythmic basis, and timing of their cycles is entrained through the influences of ambient light, physical activity, and feeding. Forced desynchronization of these systems by prolongation of a normal “day” from 24 to 28 h has been shown to cause reversal of the usual pattern of diurnal cortisol release, increases of insulin and postprandial blood glucose, and alterations in levels of epinephrine, norepinephrine, and leptin (Sheer et al. 2009). Technological advances with cultural and economic shifts encouraging round-the-clock stimulation may exacerbate or cause insomnia in susceptible individuals through desynchronization of physiological mechanisms from their otherwise endogenous rhythms. Individuals with shift-work sleep disorder, for example, have been found to have electrophysiological evidence of reduced sensory memory and hyperattention to novel sounds, compared with healthy day workers (Gumenyuk et al. 2010).

In convergence with the hyperarousal theory, it is well established that sleep disturbances including insomnia are common sequelae of traumatic stress (Spoormaker and Montgomery 2008; Charuvastra and Cloitre 2009; Pigeon et al. 2011). A review of polysomnographic studies found that individuals with post-traumatic stress disorder (PTSD) have reduced slow wave sleep (Kobayashi et al. 2007). Furthermore, it appears that pretraumatic sleep disturbance is a predictor for development of psychiatric morbidity after a traumatic event (Bryant et al. 2010). Thus, with respect to traumatic pathology as well, it appears that sleep disturbance may be not only a secondary phenomenon but possibly also a causal factor.

Fundamentally, the nature of what sleep itself “is,” has not been established with definitive consensus. A long tradition of investigation has conceptualized sleep as a global state under top–down, central regulatory control (e.g., Saper et al. 2005). This model describes competing homeostatic drives for sleep versus wakefulness and focuses on biochemical mediators of sleep including “sleep regulatory substances.” In contrast, a view of sleep focusing on synchronization of activity in local neural networks has been recently proposed (Krueger et al. 2008). In this model, local assemblies of neurons (individual cortical columns) synchronize with one another in an activity-dependent way (i.e., following a period of stimulation). Perhaps counterintuitively, some regions of the brain can be described as being in a “sleep-state” while other regions are “awake.” Global, whole-organism sleep is explained as an emergent property of the local networks.

Although the local network synchronization model does not exclude the role of metabolic factors (and pharmacological interventions) as primary initiators of local sleep states, it would appear that the model has potential to re-frame the approach to therapeutics in sleep medicine, given the physics of oscillatory synchronization (as well as the relative ease of measuring phenomena related to neural synchronization, e.g., through EEG). Therapeutic strategies that target neural oscillatory aspects of sleep, through nonpharmacological mechanisms, may be particularly attractive, in consideration of the risk of side effects and dependency associated with many pharmacological interventions for sleep disorders.

High-resolution, relational, resonance-based, electroencephalic mirroring (HIRREM™, Brain State Technologies, LLC, Scottsdale, AZ) is a noninvasive approach to enhancing neurodynamic self-regulation by giving the brain an opportunity to perceive its own oscillatory pattern. HIRREM, also known as Brainwave Optimization™, uses sound (musical tones) to reflect the brain's changing pattern of frequency-specific electrical activity back to itself. In essence, the individual is given an opportunity to “listen” to his or her own brain. HIRREM musical tones are chosen on the basis of pattern-recognition algorithms in HIRREM software. Because of the identity between the dominant EEG frequency and the frequency of the musical tone, the phenomenon of resonance occurs between the individual's brain and the musical tones. The operational theory is that neural-musical resonance may be a mechanism for autocalibration of neural networks. Because the technology does not rely on entraining the brain toward operator-defined norms for the neural energetic ratios, HIRREM is considered a procedure for autocalibration of neural oscillations. Provision of the technology does not depend on clients' active cognitive engagement.

Use of HIRREM has been anecdotally associated with amelioration of a variety of symptoms including sleep complaints (L. Gerdes, pers. comm.), and so the aim of this pilot clinical trial was to evaluate the efficacy of HIRREM for relieving symptoms of insomnia. Our primary hypothesis was that the addition of HIRREM to usual care would be superior to usual care alone, for reduction of self-reported insomnia severity.

## Methods

### Participants

This single site study was carried out in the Department of Neurology at Wake Forest Baptist Health, an academic medical center in Winston-Salem, NC. A total of 20 men and women over the age of 18 having a clinical diagnosis of insomnia and an Insomnia Severity Index (ISI) score ≥15 were recruited by physician referral and by advertisements throughout the institution. This was a pilot superiority trial with no previous randomized clinical trials of HIRREM available on which to base power calculations. Subjects were excluded if they had a history of known sleep apnea, restless legs/periodic limb movement disorder, seizure disorder, urinary problems such as benign prostate hypertrophy, severe hearing impairment, or ongoing treatment with opiates, benzodiazepines, or antipsychotic medications. Subjects were requested to abstain from using alcohol or recreational drugs during, and for 3 weeks following the HIRREM study period. Subjects were also advised not to undergo selected health care cointerventions including manual therapies during, and for 3 weeks following the HIRREM portion of the study. Additionally, participants were requested to refrain from caffeine consumption after 1:00 pm. All subjects were also instructed to continue their usual care, which was defined as whatever other medications or therapies, outside of those listed above as exclusions, that subjects were using prior to enrollment.

### Study design

**Figure 1:**
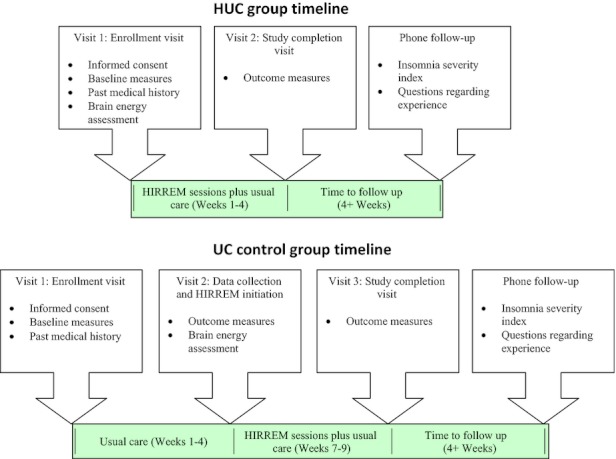
Timelines for occurrence of specific activities in the two groups (HUC and UC).

A randomized, unblinded, wait-list control, crossover, superiority study design was utilized, and the protocol was approved by the Institutional Review Board of Wake Forest School of Medicine, which did not require data safety and monitoring board oversight. The 20 subjects were randomly allocated using a blocked randomization design, with a block size of four, and a 1:1 ratio. The randomization scheme, utilizing sequentially numbered sealed envelopes containing group assignments, was created independently by a team member having no contact with the subjects, and was maintained secure by the principal investigator. Group assignments were made independent of team members enrolling the subjects. This resulted in 10 subjects being assigned to the wait-list usual care control group (UC) and 10 assigned to HIRREM plus usual care (HUC) groups. All subjects provided informed consent during an enrollment visit (V1), initial measures obtained, and past medical history obtained. During week #1, the HUC group received a HIRREM assessment and began HIRREM sessions which continued until week #4 (Fig. 1). During weeks #4 and #5, the HUC group returned for the study completion visit where posttreatment measures were obtained (V2). During weeks #5 and #6, the UC group returned for another data collection visit (V2). During week #7, the UC group had their brain energy assessments and began HIRREM sessions which lasted until week #9. During weeks #10 and #11, the UC subjects returned for study completion visits and HUC subjects were contacted for a telephone follow-up at least 4 weeks after their study completion visit. As usual care was maintained throughout the study, there was no washout period and no carryover effect needed to be calculated. There were no rules or restrictions placed on sleep hygiene or naps.

### Primary intervention

**Figure 2:**
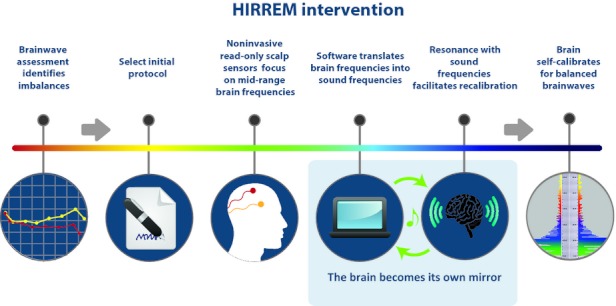
Schematic of key components of the HIRREM intervention.

The HIRREM intervention began with an initial assessment (45 min), which enabled identification of relative balance or symmetry between homologous brain regions, as well as the harmony or proportionation of energy among different frequency bands. The assessment was followed by a series of active HIRREM sessions (90 min each). The system uses unique sensors placed on the scalp according to standard International 10–20 EEG locations (Jasper 1958), and is held in place using standard EEG conductive paste. The sensors measure the frequencies (Hz) and amplitudes (μv) of brain energy overlying the major lobes. The sensors utilize embedded computer chips to filter electromagnetic interference and artifact, allowing collection of precise frequency data to enhance resolution of the functional aspects of the brain. Two recording leads, two reference leads, and one ground were used in conjunction with an EEG preamplifier. Data were recorded and viewed with a Dell Precision T3500 PC running Windows Vista, and proprietary data collection software (Brain State Technologies, LLC, Scottsdale, AZ). For the assessment, measurements were taken at homologous regions of the bilateral hemispheres (F3/F4, C3/C4, T3/T4, P3/P4, O1/O2 for both eyes closed (EC; 1 min), eyes partially open (1 min), and eyes open (EO; 1 min) conditions, while the subject was seated. For EC, and eyes partially open assessments, subjects were asked to take a deep breath and relax. For EO assessments, subjects were given standardized tasks involving numerical digit recall (F3/F4), reading silently (C3/C4), math calculations (P3/P4), listening comprehension (T3/T4), and to relax with eyes open (O1/O2). A sixth midline measurement was taken at FZ/OZ, with an EO task to count number of appearances of a specific word as they read a standardized printed passage. The reference sensors were connected at A1/A2 and linked for assessments (Fig. 2).

HIRREM sessions generally consisted of between four and eight individual HIRREM protocols, lasting between 6 and 10 min each. Protocols were intended to facilitate balance and harmony between and within brain regions. Individual protocols included up to two recording leads, two reference leads, and one ground lead using the same equipment as for the assessment. Most protocols (a combination of sensor montage and the specific software design) were two channel and recorded homologous regions of the contralateral hemispheres, but occasionally two channel, single-sided protocols or one channel protocols were used. The sensor locations and names largely corresponded to the expanded international 10-20 system; the 10-5 system (Oostenveld and Praamstra 2001).

During a protocol, and with sensors in place over the desired scalp locations, a mathematical algorithm selected the musical tone to be reflected back to the user by identifying the dominant frequency of the individual's EEG spectrum in a floating middle range, at a given instant of time. The dominant EEG frequency was then translated to a musical tone based on that frequency. The musical tone was played back to the individual through earphones, and presented binaurally with less than a 25-msec delay. Resonance between the musical tones and oscillating neural circuits was presumed to facilitate autocalibration and movement toward improved balance and harmony. Some protocols were accomplished with eyes open (rostral brain regions) and some with eyes closed (caudal brain regions).

Subjects received 8–12 HIRREM sessions, of up to 90 min per session. The number of sessions was guided by the balance and stability of the energetic pattern and neurodynamics seen during HIRREM sessions. Subjects had two HIRREM sessions in a half day, separated by a 30- to 60-min break. The majority of clients underwent four sessions in a 2-day period, and all clients completed their HIRREM sessions within 3 weeks of beginning, with most administered during the day. Each HIRREM session comprises 4–8 protocols focused on balancing specific frequencies in targeted locations on the scalp. HIRREM sessions were administered by experienced technologists who were certified in the methodology by Brain State Technologies. During sessions, subjects were encouraged to recline in a zero gravity chair (PC^6^, Human Touch, LLC, Long Beach, CA).

### Outcome measures

The primary outcome measure was the ISI (Bastien et al. 2001). All other outcomes were secondary, or exploratory. Outcome measures were obtained during the enrollment visit, post-treatment visit, and for the UC group at the repeat data collection visit (V3). Patients responded to the pencil and paper tests: ISI (primary outcome), the Center for Epidemiologic Studies Depression Scale (CES-D), the SF-36 health and well-being survey, the Medical Outcomes Survey Sleep Scale (MOS-SS), the Connor–Davidson Resilience Scale, and Visual Analogue Scales (VAS) for stress, depression, anxiety, fatigue, pain, relaxation, and overall well-being. A computerized battery of neuropsychological measures, was also administered to assess neuropsychological and psychophysiological function in multiple domains including verbal memory, visual memory, finger tapping, symbol digit coding, Stroop testing, shifting attention, and continuous performance (CNS Vital Signs, Morrisville, NC). Physiological data collected included blood pressure (BP) and a 10-min continuous recording of heart rate recording with the subject at rest. The heart rate recordings were made using the Bioharness (Biopac Systems, Inc., Goleta, CA), a noninvasive chest strap worn by the participants. The heart rate recordings included beat to beat intervals, and the data could be processed to obtain heart rate variability (HRV) data. HRV statistics which could be generated included mean, variance, standard deviation of normal to normal RR intervals (SDNN), square root of the mean squared difference of successive normal to normal RR intervals (RMS-SD), very low frequency (VLF), LF, HF, total power (TP), LF/HF, sample asymmetry, sample entropy, and coherence. All of the algorithms for computation of these parameters are derived from information or source code from the Physionet archive (Goldberger et al. 2000).

### Follow-up and safety

All outcome measures were recorded before the study began and before crossover for both groups. Only the UC group repeated all measures after the crossover intervention. Both groups had repeated ISI at a final phone follow-up at 4 or more weeks after completion of the HIRREM intervention. No adverse events or side effects were reported by any participant at any point in the study.

### Statistical analysis

All analyses were conducted using SAS 9.2 (SAS, Inc., Cary, NC). Because this is a pilot trial, no a priori power calculations were conducted prior to initiating enrollment; sample sizes were selected based on a sufficient number to estimate the treatment effect size. The primary and secondary analyses were conducted using multilevel random effects models. For the primary outcome, ISI score was modeled specifying random intercepts for participants (i.e., accounting for variance in the initial levels of insomnia across participants at baseline) with group (HUC vs. UC) and time (baseline vs. post-treatment) as fixed effects. The group × time interaction was interpreted as the differential change of the HUC group compared with the UC group. Secondary outcomes were similarly modeled, with the follow-up period added to examine the duration of change. To estimate the size of effect, Cohen's *d* was calculated for all outcome measures to index the size of the group differences in terms of within-group standard deviations (e.g., 1.2 standard deviation difference between the groups). Although arbitrary ranges, standard deviation differences ≤0.2 are often considered “small”, *d* = 0.5 are considered “medium,” and *d* > 0.80 are “large.” Descriptive statistics are presented as means (SD) or frequency counts (%) as appropriate. All point estimates of differential change are presented with 95% confidence intervals. Where appropriate, all hypothesis testing is two-tailed with *P* < 0.05 interpreted as statistical significance.

## Results

### Baseline data and subject flow

**Figure 3:**
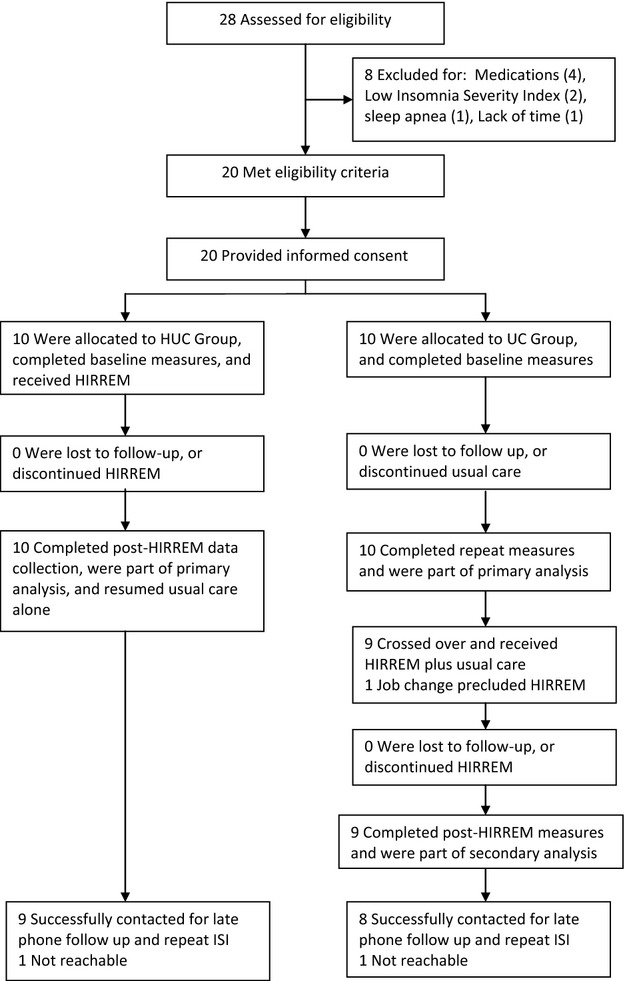
Subject recruitment and flow through the study.

A total of 28 subjects were enrolled in the study at Wake Forest Baptist Health (Fig. 3). Recruitment took place from March 1, 2011, through May 1, 2011. Twenty participants were assigned to either the wait-list UC or HUC group. Demographics and baseline characteristics (Table 1) were not statistically different between the two groups. There were slightly more comorbidities noted in the HUC group (Table 2). Antidepressants were used by three subjects in the HUC group, and one in the UC group. All patients continued their usual care throughout the course of the study; HIRREM was added to usual care during the primary intervention epoch. All subjects completed the primary intervention period, and primary data collection visits. All 10 participants in the HUC group received HIRREM (mean of 10.3 sessions) and nine of 10 UC subjects subsequently received HIRREM after crossover. One in the UC group had a job change and the schedule prevented further participation. One subject from each group receiving HIRREM was not available for the late telephone follow-up.

**Table 1:** Baseline demographics

	HUC intervention group (SD)	UC control group (SD)
*N*	10	10
Mean age	41.3 (17.5)	49.5 (8.1)
Women/Men	8/2	6/4
Ethnicity	9/10 Caucasian	10/10 Caucasian
Mean baseline ISI	18.75 (2.7)	18.9 (3.2)
CES-D	17.1 (11.1)	12.6 (7.1)
SF-36: General health	72 (28.0)	69 (20.4)
Systolic blood pressure	115.7 (9.6)	116.2 (9.4)
Heart rate	74.4 (12.8)	71.6 (9.5)

HUC, HIRREM plus usual care; UC, usual care; ISI, Insomnia Severity Index; CES-D, Center for Epidemiologic Studies Depression Scale.

**Table 2:** Self-reported comorbidities

Medical condition/Comorbidity	HUC intervention group	UC control group
Hypertension	2	2
Hyperlipidemia	3	1
Headaches/Migraine	3	0
Stress/Anxiety disorder	2	1
Depression	3	2
Trauma/TBI	1	1

HUC, HIRREM plus usual care; UC, usual care; TBI, traumatic brain injury.

### Primary outcome

**Figure 4:**
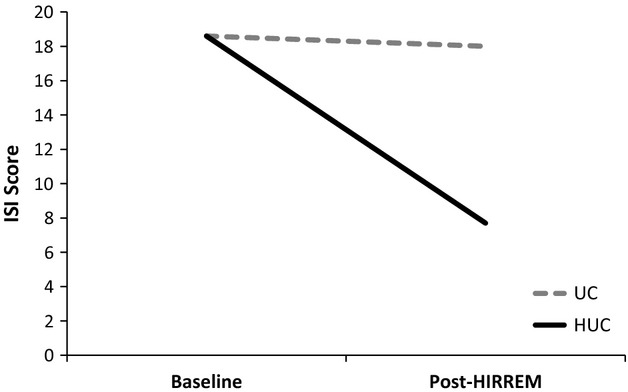
Baseline and post-HIRREM Insomnia Severity Index (ISI) scores for usual care (UC) and HIRREM plus usual care (HUC) groups. Differential change: −10.3 (95% CI: −13.7 to −6.9), *P* < 0.0001.

Mean baseline ISI for each group was identical, at the enrollment visit (mean = 18.6, *P* = 1.0). The primary outcome for the study, analysis for differential change in the ISI at V2 (Fig. 4), showed a statistically significant drop of 10.3 points (−13.7 to −6.9; *P* < 0.0001). Standard effect size (Cohen's *d*) was 2.68 for change in ISI.

### Secondary outcomes

**Figure 5:**
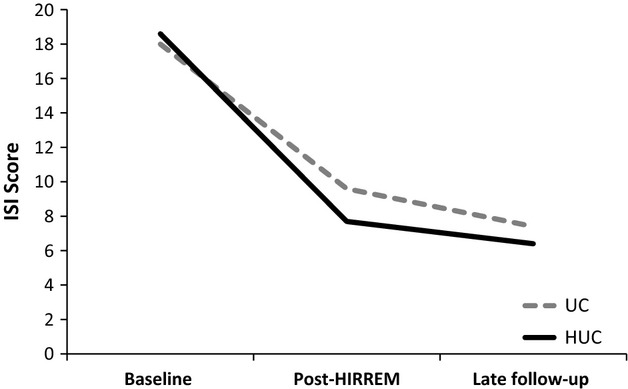
Baseline to post-HIRREM changes in Insomnia Severity Index (ISI) scores for usual care (UC) and HIRREM plus usual care (HUC) groups after cross-over, with 4- to 6-week late follow-up ISI scores.

The UC group was then offered crossover to receive HIRREM. There was no statistical difference for analysis of differential change in the ISI following HIRREM intervention between the HUC group and the crossover UC group. The ISI was also administered at a telephone follow-up at least 4 weeks following completion of the HIRREM intervention. The improvement in insomnia symptoms reported following completion of the HIRREM sessions persisted through that period (Fig. 5).

Considering clinical threshold correlates for insomnia, based on the differential change in mean ISI, the HUC group improved to just under the cut point for subthreshold insomnia category, while the UC group remained in the moderate insomnia category (Table 3). As a way to consider clinically relevant changes for individual subjects, the number of subjects in each category, before and after each study epoch, shows that 9/10 in the UC group remained in the moderate-to-severe insomnia category, while 9/10 in the HUC group moved to the no insomnia or subthreshold categories following HIRREM. Following crossover and receipt of HIRREM, 6/9 in the UC group also improved to no insomnia or subthreshold insomnia, and the effects persisted with late follow-up after HIRREM for both groups.

**Table 3:** Changes in clinical category for insomnia after HIRREM based on ISI scores

	HUC intervention group			UC control group			
		
Clinical category by ISI score	Pre	Post	Late phone F/U	Pre	Post	After crossover HIRREM	Late phone F/U
No clinically significant insomnia (0–7)	0	5	5	0	0	2	5
Subthreshold insomnia (8–14)	0	4	4	0	1	4	1
Moderate insomnia (15–21)	9	1	0	7	8	3	3
Severe insomnia (22–28)	1	0	0	3	1	0	0

HIRREM, high-resolution, relational, resonance-based, electroencephalic mirroring; HUC, HIRREM plus usual care; UC, usual care; ISI, Insomnia Severity Index.

Differential change in the CES-D score during the primary intervention period reached statistical significance with a drop of 8.8 points (−17.5 to −0.1; *P* = 0.047). Differential change was not statistically significant for the total SF-36 score, which increased by 4.0 (−6.8 to 14.8; *P* = 0.446), but there were small effect sizes for some components of the SF-36, with effect size values ranging from 0.07 for physical function to 0.58 for energy and fatigue. There were also no statistically significant changes for the neurocognitive measures, although several domains, psychomotor speed (0.38), neurocognitive index (0.24), and complex attention (0.22) showed small effect sizes. Due to the small sample size, there was inadequate power for analysis of other secondary and exploratory outcome measures. Poor technical quality of recordings precluded analysis of HRV measures.

**Figure 6:**
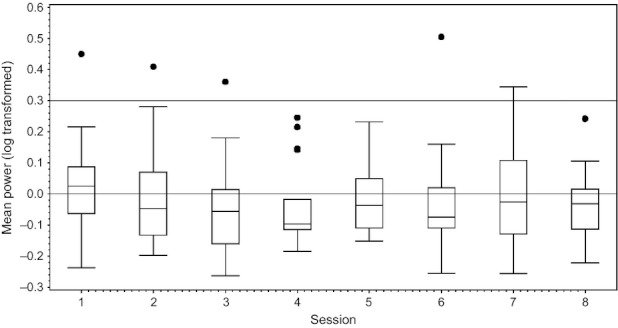
Tukey box plot of mean power (log transformed) in the high-frequency (23–36 Hz, “80”) range at the temporal locations (T3 and T4, averaged together), over the course of eight HIRREM sessions, *n* = 19 subjects.

Exploratory analysis of changes in the brain pattern following HIRREM, for all those who received HIRREM (*n* = 19), suggested that there was a decrease in the overall power in high frequencies (23–36 Hz) at the temporal lobes in the T3/T4 location (Fig. 6), over the course of the required minimum of eight HIRREM sessions. The median for log transformed mean power values showed a steady decline over the first four HIRREM sessions. The median for high-frequency power then appeared to oscillate, seemingly around a lower set point, for the remaining sessions.

## Discussion

This study represents the first use of HIRREM in a randomized clinical trial. HIRREM was a feasible, effective intervention for such an outpatient population, and appeared both safe and well tolerated. Based on our primary outcome measure of differential change in the ISI score, as an addition to usual care, use of HIRREM was associated with an improvement of insomnia symptoms in this study population of subjects with moderate-to-severe insomnia. The standard effect size suggested that as applied during this study, HIRREM had a strong effect. Based on telephone follow-up done at least 4 weeks following HIRREM, the improvement in ISI persisted. When crossed over to receive HIRREM, those in the UC group showed similar differential change in the ISI, with similar persistence of the effect on late telephone follow-up. When considered in light of clinical correlates with the ISI, nine of 10 subjects in the HUC group moved to an ISI score in the no insomnia or subthreshold insomnia categories. Following crossover, and receipt of HIRREM intervention, six of nine in the UC group also moved to no insomnia or subthreshold insomnia categories, suggesting clinically relevant changes in this population following HIRREM.

Among other secondary outcomes, differential change (improvement) in the CES-D measure of depression just reached statistical significance, while there was no significant change in formal measures of overall health and well-being (SF-36), or neurocognitive function, as measured by a computerized neurocognitive battery. Depression is closely intertwined with insomnia, and future studies may help elucidate whether improvement in either sleep or mood appears to be causal to improvement in the other. The small sample size and the specific measures used do not allow identification a specific effect for depression.

Although the exact mechanism of action of HIRREM has not yet been confirmed, the secondary finding of a decrease in overall high-frequency power in the temporal lobes may provide some insights. Exploratory analysis of brain changes was focused on the temporal lobes based on the supposition that temporal lobe activity may reflect autonomic functioning. Craig (2005) has reported a neuroanatomical basis for lateralization of autonomic nervous system management by the right and left insula for the sympathetic and parasympathetic divisions, respectively. Thus, increased overall power in the temporal lobes, if reflective of activation of autonomic functioning, is consistent with the hyperarousal theory regarding the underlying mechanism for insomnia. Quieting of high-frequency power in the temporal lobes could be understood as mitigating an underlying driver of insomnia.

### Limitations

The limitations of this study include a small sample size, as well as the use of a wait-list usual care control group rather than an active control, or sham-placebo group. Because the study design entailed usual care for the control group, without blinding as to the intervention, it is not possible to rule out placebo or expectation effects as contributors to the improvements associated with the HIRREM intervention. HIRREM, like other interventions which entail social interaction and relaxation induction, may facilitate improvements not only through auditory tonal mirroring of dominant electroencephalic frequencies but also through nonspecific mechanisms. Placebo biofeedback interventions, for example, have in some cases been shown to offer benefits comparable to true biofeedback (Nicassio et al. 1982; Hunyor et al. 1997). Nonetheless other studies have reported that true biofeedback is more efficacious than placebo biofeedback (Henderson et al. 1998; Armagan et al. 2003; Becerra et al. 2006; Rao et al. 2007; Basta et al. 2011). The degree of improvement, and the standard effect size, coupled with persistence of benefit for at least 4 weeks following completion of HIRREM suggests the presence of a real change. In addition, subjects in both groups continued their usual care throughout the course of the study. It is unclear whether HIRREM alone would achieve the results observed or if combination is necessary. Placebo-controlled studies of HIRREM are warranted, and future studies should include physiological outcomes and follow-up to evaluate persistence of effect.

## Conclusion

In this pilot clinical trial, the use of HIRREM in subjects with insomnia was feasible and effective and was safe and well tolerated. Based on differential change for a subjective clinical insomnia outcome measure, HIRREM improved insomnia compared with continuation of usual care alone. This appeared to be a strong effect based on the standard effect size, and the effect persisted for at least 4 weeks following HIRREM. The CES-D also showed improvement. Exploratory analysis suggested changes in brain pattern having relevance to the hyperarousal theory of insomnia, with potential implications for understanding the mechanisms of HIRREM for individuals with insomnia. This study suggests a need for additional controlled clinical trials to both confirm the effect and further explore possible mechanisms of action.
